# Exploring the Antimicrobial Potential of Hallachrome, a Defensive Anthraquinone from the Marine Worm *Halla parthenopeia* (Polychaeta)

**DOI:** 10.3390/md22090380

**Published:** 2024-08-24

**Authors:** Anita Ferri, Roberto Simonini, Carla Sabia, Ramona Iseppi

**Affiliations:** 1 Department of Chemical and Geological Sciences, University of Modena and Reggio Emilia, Via Giuseppe Campi 103, 41125 Modena, MO, Italy; 224420@studenti.unimore.it; 2 Department of Life Sciences, University of Modena and Reggio Emilia, Via Giuseppe Campi 213/D, 41125 Modena, MO, Italy; carla.sabia@unimore.it (C.S.); ramona.iseppi@unimore.it (R.I.)

**Keywords:** anthraquinone, mucus, defensive, *Candida albicans*, *Staphylococcus aureus*, *Staphylococcus epidermidis*, *Enteroccocus faecalis*, *Pseudomonas aeruginosa*, *Escherichia coli*

## Abstract

Antimicrobial resistance is a critical global health issue, with rising resistance among bacteria and fungi. Marine organisms have emerged as promising, but underexplored, sources of new antimicrobial agents. Among them, marine polychaetes, such as *Halla parthenopeia*, which possess chemical defenses, could attract significant research interest. This study explores the antimicrobial properties of hallachrome, a unique anthraquinone found in the purple mucus of *H. parthenopeia*, against Gram-negative bacteria (*Escherichia coli* ATCC 25922, *Pseudomonas aeruginosa* ATCC 9027), Gram-positive bacteria (*Enterococcus faecalis* ATCC 29212, *Staphylococcus aureus* ATCC 6538, *Staphylococcus epidermidis* ATCC 12228), and the most common human fungal pathogen *Candida albicans* ATCC 10231. Antibacterial susceptibility testing revealed that Gram-negative bacteria were not inhibited by hallachrome at concentrations ≤2 mM. However, Gram-positive bacteria showed significant growth inhibition at 0.12–0.25 mM, while *C. albicans* was inhibited at 0.06 mM. Time-kill studies demonstrated dose-dependent growth inhibition of susceptible strains by hallachrome, which exerted its effect by altering the membrane permeability of *C. albicans*, *E. faecalis*, and *S. epidermidis* after 6 h and *S. aureus* after 24 h. Additionally, hallachrome significantly reduced biofilm formation and mature biofilm in *S. aureus*, *E. faecalis*, and *C. albicans*. Additionally, it inhibited hyphal growth in *C. albicans*. These findings highlight hallachrome’s potential as a novel antimicrobial agent, deserving further exploration for clinical experimentation.

## 1. Introduction

Antimicrobial resistance stands as one of the most pressing global health concerns of our time [1]. The escalation, emergence, and dissemination of resistance among various microorganisms, including bacteria, fungi, viruses, and parasites, has become a high-impact concern with considerable clinical and economic imprint, as recently highlighted in the 2019 document by the WHO [2]. This phenomenon represents a significant threat to both community and hospital settings, accentuating illness, increasing the frequency of nosocomial infections, and raising mortality rates worldwide [3,4,5]. The indiscriminate and excessive use of antibiotics in both the human and veterinary fields has played a crucial role in increasing the percentage of antimicrobial resistance in the main pathogens under surveillance (*Staphylococcus aureus*, *Enterococcus faecalis*, *Enterococcus faecium*, *Escherichia coli*, etc.) [6,7,8]. *Candida albicans* is the predominant opportunistic fungal pathogen in humans, causing superficial and systemic infections. The incidence of invasive *C. albicans* infections has risen due to the widespread use of limited antifungal options [9,10,11]. One of the major *C. albicans* virulence characteristics is its capacity to form biofilms, which, adhering to both biological and non-biological surfaces, further complicate clinical treatment strategies [12,13]. In particular, the virulence factor associated with *C. albicans* biofilm is hyphal morphology. The transition from the yeast-to-hyphal phase plays a role in the pathogenicity of *C. albicans*, given that hyphae can attach to and damage host cells and tissues and escape host immune defenses [14].

Natural substances represent a significant segment of the pharmaceutical market, with most antibacterial agents used clinically as naturally derived or resembling natural products [15]. Historically, the search for new natural antimicrobial agents has predominantly focused on terrestrial environments, particularly soil. However, the vast and largely unexplored marine habitats harbor immense potential for the discovery of novel antimicrobial compounds [16]. The intricate ecological interplay within marine ecosystems offers a rich reservoir of bioactive molecules, including peptides, terpenoids, polysaccharides, and alkaloids, derived from a diverse array of organisms such as bacteria, fungi, seaweeds, and sponges [17]. This underexploited resource presents a promising way for the development of urgently needed antibiotics as well as other biotechnological applications.

Among marine organisms, polychaetes, a diverse group of aquatic invertebrates, have gained attention as potential sources of bioactive compounds. These organisms, abundant in marine environments ranging from intertidal zones to deep-sea vents, possess intricate defense mechanisms against pathogens, including the synthesis of active molecules [18,19]. Even if research into the bioactive compounds produced by polychaetes is still at the beginning, promising antimicrobial candidates have already been identified, underscoring the importance of further exploration in this field [20,21,22]. Among the compounds with antimicrobial potential, there is Hedistin, produced by specialized cells within its coelomic fluid of *Hediste* (Nereis) *diversicolor*. This compound exhibits bacteriostatic activity, displaying the organism’s innate defense mechanisms against microbial pathogens [23,24]. Similarly, *Glycera dibranchiata* secretes antimicrobial factors in its coelomic fluid, suggesting an adaptive response to bacterial threats [25]. There are also non-proteinaceous antimicrobials from polychaetes, such as thelepin (produced by *Thelepus setosus*), which was found to have similar characteristics to antimycotics obtained from *Penicillium* fungi [26]. Additionally, crude mucus secretions from certain polychaetes possess antibacterial and antifungal properties, even if their precise constituents remain unknown [27]. One notable polychaete species, *Halla parthenopeia*, has been proposed as a promising candidate for bioprospecting efforts. Inhabiting the soft bottoms of the Mediterranean Sea, *H. parthenopeia* produces a purple mucus containing a unique compound called hallachrome. In fact, it is a rare 1–2 anthraquinone, which is the only one known to date isolated from an invertebrate [28]. Hallachrome (HC) exhibits potent toxicity against a range of organisms, including bacteria, protozoans, rotifers, and crustaceans [21]. This compound is secreted in response to mechanical stress, possibly aiding in wound infection prevention in injured worms [29,30]. Hallachrome belongs to anthraquinones (AQs), a diverse class of natural products that have garnered interest for their myriad health benefits, including anticancer, antibacterial, and antifungal properties [31]. Derived mainly from plants, AQs like emodin, alizarin, and purpurin have demonstrated antibacterial and antibiofilm activities against fungal and bacterial pathogens [32]. Their effectiveness is influenced by their chemical structure, particularly substituent groups, with greater polarity correlating with increased antibacterial potency [33,34]. Hallachrome may represent a valuable target for exploration in drug discovery also because of the availability of procedures for the long-term maintaining of the *H. parthenopeia* under laboratory conditions [29] and the purification of HC directly from the purple mucus [21], which allow for obtaining the adequate amount of substance without sacrificing the worms [21,29].

The present study aims to assess the antimicrobial activity of the hallachrome in vitro against bacterial strains belonging to Gram-negative bacteria (*Escherichia coli* ATCC 25922, *Pseudomonas aeruginosa* ATCC 9027) and Gram-positive bacteria (*Enterococcus faecalis* ATCC 29212, *Staphylococcus aureus* ATCC 6538, and *Staphylococcus epidermidis* ATCC 12228), as well as the human fungal pathogen *Candida albicans* ATCC 10231. After a preliminary analysis to identify susceptible strains, further investigations were carried out to elucidate the mode of action of HC, including its ability to damage the cell membranes and interfere with the virulence factors (e.g., hyphae formation) of the tested strains. Lastly, the ability of HC to inhibit biofilm formation and disrupt mature biofilm was evaluated.

## 2. Results

### 2.1. Antibacterial Susceptibility Testing and MIC Determination

The growth of tested Gram-negative bacteria (*E. coli* and *P. aeruginosa*) was not inhibited by HC concentrations of 2 mM or lower; thus, they are not considered susceptible. For the tested Gram-positive bacteria, there is an inhibition of growth greater than 85% at concentrations between 0.12 mM and 0.25 mM. The lowest MIC value (0.06 mM) was observed for *C. albicans*. All the MIC values and the corresponding inhibition rates are reported in Table 1.

### 2.2. Time Kill Studies

**Figure 1:**
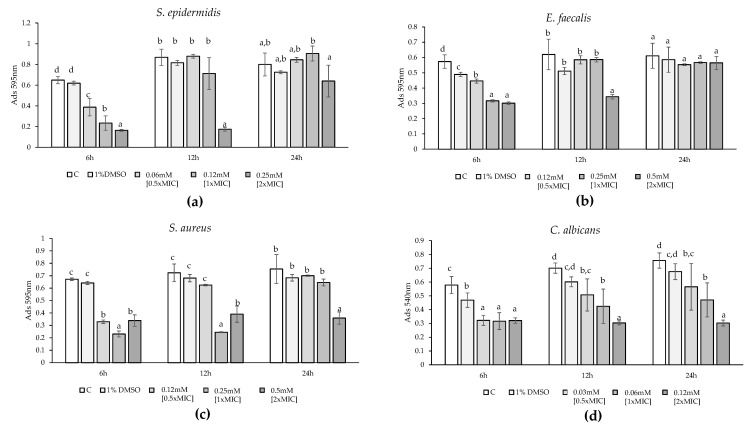
Effect of treatment with HC at 0.5×MIC, 1×MIC, and 2×MIC on growth of *S. epidermidis* (**a**), *E. faecalis* (**b**), *S. aureus* (**c**), and *C. albicans* (**d**) after 6, 12, and 24 h compared with untreated (Control) and treated with solvent (1% DMSO). Significant differences (*p*-value < 0.05) among the growth rate are reported and indicated with different letters.

**Figure 2:**
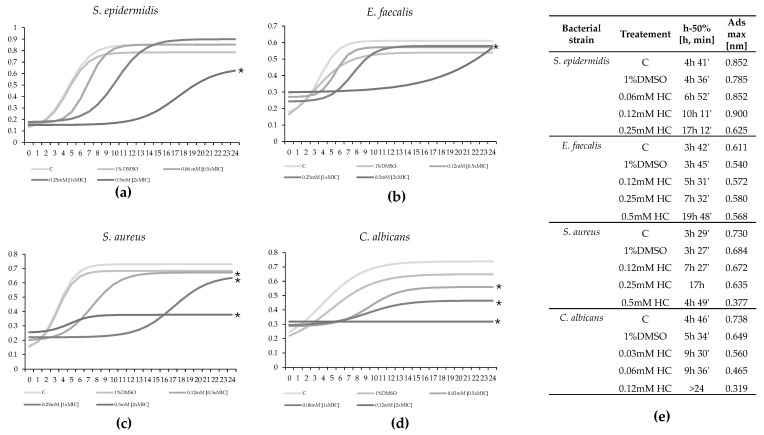
Sigmoid model of the effect of HC treatment at 0.5×MIC, 1×MIC, and 2×MIC on the growth of *S. epidermidis* (**a**), *E. faecalis* (**b**), *S. aureus* (**c**), and *C. albicans* (**d**) during 24 h exposure, compared to untreated (Control) and solvent-treated (1% DMSO) controls. Differences between treatments were highlighted in terms of the time required to reach 50% growth for each treatment and the maximum growth achieved after 24 h (**e**). The asterisk (*) indicates conditions that differ significantly from the control (C) (*p*-value < 0.001) by comparing the area under the curve (AUC) for the raw data kinetic curves.

The bacterial growth of susceptible strains (*S. aureus*, *S. epidermidis*, *E. faecalis*, and *C. albicans*) treated with DMSO, 0.5×MIC, 1×MIC, and 2×MIC was evaluated at 37 °C or 30 °C by measuring optical density (OD) at 595 nm and at 540 nm only for *C. albicans* at 1 h intervals over 24 h. The time points analyzed using ANOVA and Tukey’s test were 6 h, 12 h, and 24 h. *S. epidermidis* growth was significantly inhibited after 6 h by all three tested concentrations (F = 53.65, *p*-value < 0.0001) in a dose-dependent manner, with reductions of 41% for 0.5×MIC, 64% for 1×MIC, and 75% for 2×MIC. At 12 h, only 2×MIC showed a significant reduction in bacterial growth (−80%), while after 24 h, the treatments did not differ significantly from the controls (Figure 1a). Modeling the growth curves of the various treatments as sigmoid curves showed a dose-dependent delay in growth. The control reached 50% of maximum growth before 5 h, while 0.5×MIC, 1×MIC, and 2×MIC reached it at 7 h, 10 h, and 17 h, respectively (Figure 2a,e). *E. faecalis* growth was significantly inhibited at 6 h by all three concentrations (F = 53.65, *p*-value < 0.0001) with dose-dependent reductions of 22%, 44%, and 47% for 0.5×MIC, 1×MIC, and 2×MIC, respectively. At 12 h, 0.5×MIC did not significantly reduce growth, but 1×MIC and 2×MIC reduced it by over 40%. At 24 h, only 2×MIC had a significant effect (−45%), while the treatments did not differ significantly from the controls (Figure 1b). Sigmoid modeling showed a dose-dependent delay in growth. The control reached 50% of maximum growth before 4 h, while 0.5×MIC and 1×MIC reached it after 5 h 30 and 7 h 30, respectively. For 2×MIC, 50% of the growth reached after 24 h occurred at 19 h without approaching the stationary phase (Figure 2b,e). *S. aureus* growth was significantly inhibited at 6 h by all three concentrations (F = 53.65, *p*-value < 0.0001), with reductions ranging from 50% to 60%. At 12 h, 0.5×MIC did not significantly reduce growth, but 1×MIC and 2×MIC resulted in over 40% reduction. At 24 h, only 2×MIC had a significant effect (−52%) (Figure 1c). Sigmoid modeling showed a dose-dependent delay in growth. The control reached 50% of maximum growth before 4 h, while 0.5×MIC and 1×MIC reached it after 7 h and 17 h, respectively, and total growth with 2×MIC was much lower than the control (Figure 2c,e). *C. albicans* growth was significantly inhibited at 6 h by all tested concentrations (F = 53.65, *p*-value < 0.0001), with a 55% reduction for all concentrations. At 12 h, there was a significant, dose-dependent reduction in growth compared to the control (−28% for 0.5×MIC; −39% for 1×MIC; −57% for 2×MIC). At 24 h, the reductions were 25%, 38%, and 60% for 0.5×MIC, 1×MIC, and 2×MIC, respectively (Figure 1d). Sigmoid modeling showed a delay in growth, with controls reaching 50% of maximum growth before 6 h, while 0.5×MIC and 1×MIC reached it after 9 h. For 2×MIC, no relative growth was observed compared to the beginning of the experiment (Figure 2d,e).

### 2.3. Determination of Membrane Permeability Alteration (Cristal Violet Assay)

**Figure 3:**
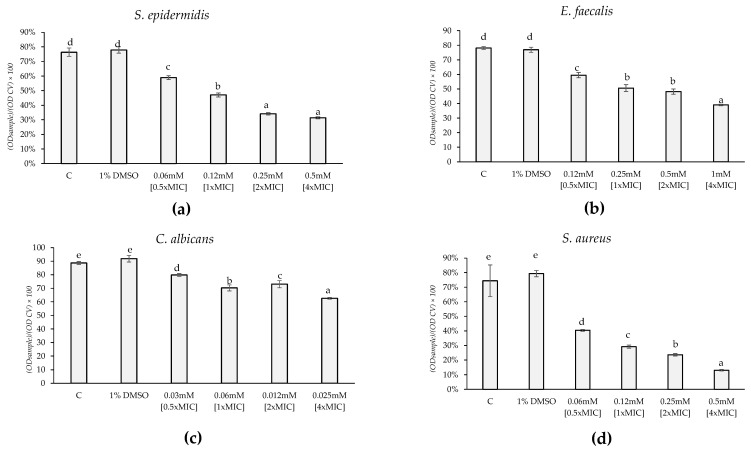
Effect of treatment with HC at 0.5×MIC, 1×MIC, 2×MIC, and 4×MIC on membrane permeability of *S. epidermidis* (**a**), *E. faecalis* (**b**), and *C. albicans* (**c**) after 6 h of treatment and of *S. aureus* (**d**) after 24 h compared with not treated and treated with solvent (1% DMSO). Lower is the (OD sample)/(OD CV) × 100; higher is the membrane permeability effect. Significant differences (*p*-value < 0.05) among the permeability are reported and indicated with different letters.

The alteration of membrane permeability was assessed using a crystal violet assay by adding HC to bacterial suspensions at concentrations of 0.5×MIC, 1×MIC, 2×MIC, and 4×MIC for 30 min, 6 h, and 24 h. The results were expressed as the percentage of crystal violet (CV) remaining in the supernatant for each bacterial treatment compared to a pure CV solution used as a control. A lower percentage of CV remaining in the supernatant indicates a greater membrane permeabilization effect. For all microorganisms tested, none of the HC concentrations resulted in significant membrane permeabilization after 30 min of exposure. However, for *C. albicans*, *E. faecalis*, and *S. epidermidis*, HC induced membrane permeabilization after 6 h of exposure (F = 542.2; *p*-value < 0.0001; F = 297.8; *p*-value < 0.0001; F = 686.5; *p*-value < 0.0001, respectively) (Figure 3). Regarding *S. epidermidis*, there was a significant increase in permeability of 23%, 38%, 55%, and 59% for 0.5×MIC, 1×MIC, 2×MIC, and 4×MIC, respectively (Figure 3a). For *E. faecalis*, the HC led to a dose-dependent increase in permeability of 24%, 35%, 38%, and 50% for 0.5×MIC, 1×MIC, 2×MIC, and 4×MIC, respectively (Figure 3b). Lastly, concerning *C. albicans*, there was a significant increase in permeability of 10%, 20%, 17%, and 29% for 0.5×MIC, 1×MIC, 2×MIC, and 4×MIC, respectively (Figure 3c). For *S. aureus*, the effect on membrane permeability was not significant at 30 min and 6 h of exposure but became significant after 24 h of exposure (F = 456.6; *p*-value < 0.0001). Specifically, the 1×MIC caused a dose-dependent increase in permeability of 38%, 56%, 69%, and 79% for 0.5×MIC, 1×MIC, 2×MIC, and 4×MIC, respectively (Figure 3d). Overall, these results indicate that HC’s ability to permeabilize bacterial membranes is both time- and dose-dependent, with significant effects observed at longer exposure times and higher concentrations.

### 2.4. HC Activity on Hyphae Formation, Biofilm Formation, and Mature Biofilm

#### 2.4.1. Hyphae Formation

**Figure 4:**
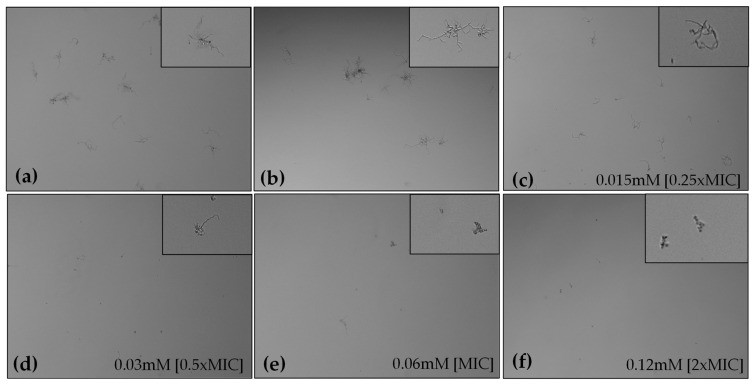
Effect of 6 h treatment with HC at 0.25×MIC (**c**), 0.5×MIC (**d**), 1×MIC (**e**) and 2×MIC (**f**), on hyphal growth of *C. albicans* compared with untreated (**a**) and treated with 1% DMSO (**b**). Details in insets.

**Figure 5:**
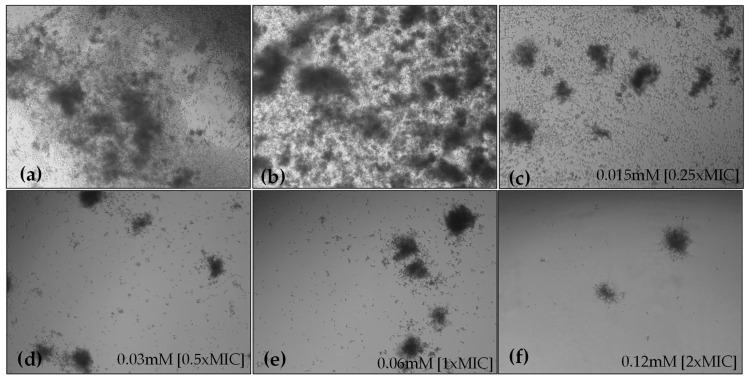
Effect of 24 h treatment with HC at 0.25×MIC (**c**), 0.5×MIC (**d**), 1×MIC (**e**) and 2×MIC (**f**) on hyphal growth of *C. albicans* compared with treated (**a**) and treated with 1% DMSO (**b**).

Microscopic observations of HC-treated fungal cells revealed that HC reduced hyphal lengths and inhibited the yeast-to-hyphae transition in a concentration-dependent manner. After 6 h of incubation, the non-treated groups showed extensive hyphal growth, whereas in the presence of HC, minimal hyphal development was observed, with cells primarily in the yeast and pseudohyphal phases (Figure 4). Microscopic observations of the 24-h *C. albicans* culture showed that HC, in addition to reducing biofilm formation, inhibited hyphal formation in liquid media (Figure 5).

#### 2.4.2. Biofilm Formation

**Figure 6:**
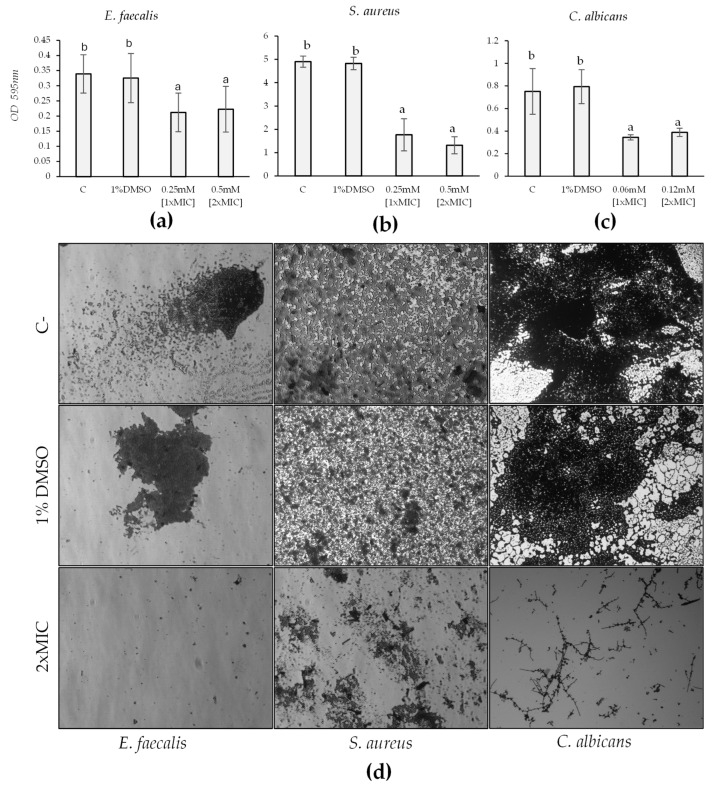
Effect of HC at 0.5×MIC, 1×MIC, and 2×MIC on biofilm formation of *E. faecalis* (**a**), *S. aureus* (**b**), and *C. albicans* (**c**) compared with untreated and treated with 1% DMSO. Significant differences (*p*-value < 0.05) among the inhibitions of biofilm formation are reported and indicated with different letters. The effect of 2×MIC on biofilm formation compared to the control was also evident under microscopic visualization (**d**).

To test the effect of HC on biofilm formation and mature biofilm, we selected strains that were good biofilm producers: *S. aureus*, *C. albicans*, and *E. faecalis*. *S. epidermidis* was excluded from this part of the study as it did not produce significant biofilm. The effect of HC on biofilm formation was evaluated by exposing the bacteria to 1×MIC and 2×MIC for 24 h after an initial 6-hour incubation without the compound. For *C. albicans*, *S. aureus*, and *E. faecalis*, there was a significant reduction in biofilm formation following exposure to HC (F = 15.28, *p*-value *<* 0.001; F = 128.6, *p*-value *<* 0.001; F = 7.42, *p*-value < 0.001) at both 1×MIC and 2×MIC. In all these observations, the effect was not dose-dependent, as there was no significant difference between the effects of 1×MIC and 2×MIC. Specifically, for *E. faecalis*, the reduction in biofilm formation was 37% for the 1×MIC value and 34% for 2×MIC value (Figure 6a). Regarding *C. albicans*, the decrease in biofilm formation was 54% and 48%, respectively (Figure 6b). Lastly, concerning *S. aureus*, the decrease was 63% and 73% for the 1×MIC and 2×MIC values, respectively (Figure 6c). Microscopic investigation validated these results obtained through spectrophotometric readings (Figure 6d).

#### 2.4.3. Mature Biofilms

**Figure 7:**
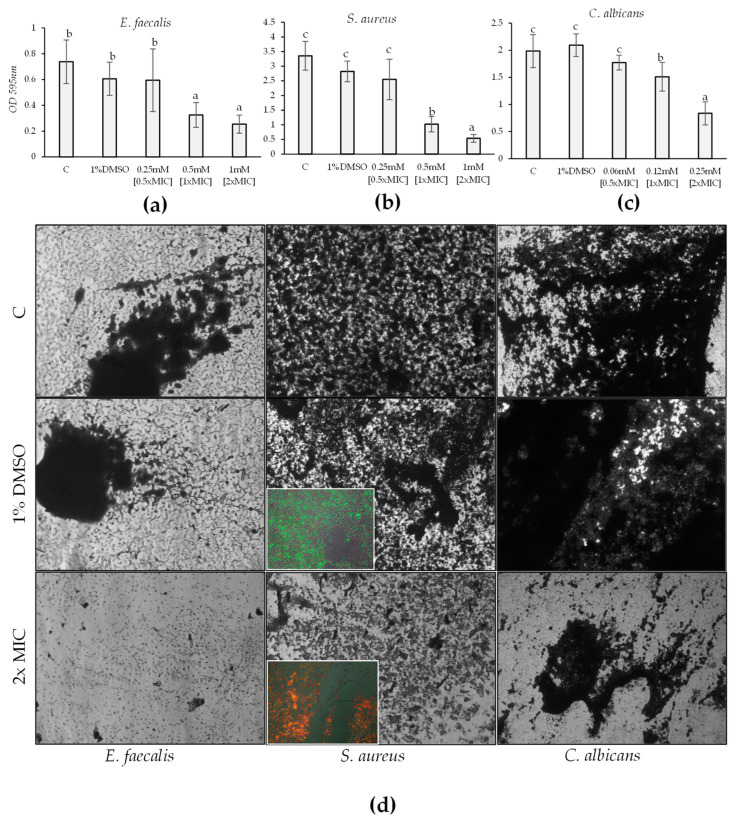
Effect of 24 h treatment with HC at 0.5×MIC, 1×MIC, and 2×MIC on mature biofilm (24 h old) of *E. faecalis* (**a**), *S. aureus* (**b**)*,* and *C. albicans* (**c**) compared with not treated and treated with 1% DMSO. Significant differences (*p*-value < 0.05) among the biofilm eradication are reported and indicated with different letters. The effect of 2×MIC on mature biofilm compared to the control was also evident under microscopic visualization (**d**). Fluorescence microscopy images to determinate the activity of HC on mature biofilm ((**d**), insets): green spots (live bacteria), yellow spots (overlapping live and dead bacteria) and red spots (dead bacteria) can be observed.

The effect of HC on mature biofilms was assessed. Regarding *C. albicans*, *S. aureus*, and *E. faecalis*, a significant reduction in mature biofilm following exposure to HC (F = 37.93, *p*-value < 0.001; F = 24.1, *p*-value < 0.001; F = 10.19, *p*-value < 0.001) was obtained. For all three tested strains, 1×MIC alone was not sufficient to reduce mature biofilms significantly, whereas 2×MIC and 4×MIC were effective. The effect was dose-dependent, with a significant difference between the effects of 2×MIC and 4×MIC. Specifically, for *E. faecalis*, 2×MIC resulted in a 56% reduction and 4×MIC in a 65% reduction; for *C. albicans*, the reductions were 24% and 57%; for *S. aureus*, they were 70% and 84% (Figure 7a–c). Microscopic investigation validated these results obtained through spectrophotometric readings (Figure 7d). Using the marker 5(6)-carboxyfluorescein diacetate (cFDA), which stains live bacteria, and propidium iodide (PI), which stains dead bacteria, from the “live/dead cells stain kit” on mature biofilm of *S. aureus* (control and treated with 2×MIC), a reduction in the viability of the mature biofilm exposed to HC compared to the control was observed. In fact, Figure 7d inset shows that most of *S. aureus* treated at 2×MIC are dead (red). Yet, upon magnification, green spots (live bacteria) and yellow spots (overlapping live and dead bacteria) can be observed, suggesting a bacteriostatic rather than a bactericidal effect.

## 3. Discussion

The antimicrobial effect of Hallachrome was evaluated against Gram-negative bacteria (*E. coli*, *p. aeruginosa*), Gram-positive bacteria (*S. aureus*, *S. epidermidis*, and *E. faecalis*), and the human fungal pathogen *C. albicans*. In this study, Hallachrome’s activity was tested only on classified strains due to technical limitations. Clinically isolated strains may respond very differently from standard ones, highlighting the need for further investigations.

The most susceptible strains were *S. aureus*, *S. epidermidis*, *E. faecalis*, and *C. albicans*, with MIC values of 0.25 mM (67 mg/L), 0.12 mM (33.5 mg/L), 0.25 mM (67 mg/L), and 0.06 mM (16.7 mg/L), respectively. These inhibitory concentrations are comparable to those reported for other naturally derived anthraquinones. The growth of *S. aureus* is inhibited at 12 mg/L by rhein, 31.25 mg/L by 1,8-Dihydroxy-4,5-dinitroanthraquinone (DHDNA), 83 mg/L by Symploquinone, and 125 mg/L by 1,5,7-trihydroxy-3-hydroxy methyl anthraquinone [8,35,36,37]. *S. epidermidis* is inhibited at 15.62 mg/L by purpurin and at 62.5 mg/L by 1,5,7-trihydroxy-3-hydroxy methyl anthraquinone [37,38]. *E. faecalis* is inhibited at 62.5 mg/L by DHDNA, 5.44 mg/L by solanrubiellin A, 125 μg/mL by 1,5,7-trihydroxy-3-hydroxy methyl anthraquinone, and 64 mg/L by pleosporone [8,37,39,40]. *C. albicans* is inhibited at 12.5 mg/L by emodin, 5.12 mg/L by purpurin, and 64 mg/L by pleosporone [40,41,42]. In contrast, Gram-negative bacteria were not susceptible to HC, as they were not inhibited at concentrations exceeding 2 mM. This is not unusual, as other anthraquinones active against Gram-positive bacteria are often ineffective against Gram-negative bacteria, even at high concentrations. For instance, *E. coli* and *P. aeruginosa* are not inhibited by alizarin at 100 μg/mL, nor by DHDNA or emodin, which are effective against Gram-positive bacteria but inactive against *E. coli* [8,38,43].

The effects of HC on the growth kinetics of susceptible bacterial strains were studied to determine whether its impact is bacteriostatic or bactericidal. Over 24 h of exposure at concentrations of 0.5×MIC, 1×MIC, and 2×MIC, HC induced a concentration-dependent delay in bacterial growth compared to controls. Exposure to 0.5×MIC and 1×MIC only delayed the time to reach maximum growth, but by the end of the 24 h period, the bacteria achieved the same growth level as the controls. In contrast, exposure to 2×MIC resulted in a significant reduction in the maximum growth rate of each susceptible strain, demonstrating a pronounced bacteriostatic effect, particularly for *C. albicans*, where the bacterial count remained essentially unchanged from the initial count. Similar bacteriostatic effects for *E. faecalis*, *S. aureus*, and *S. epidermidis* could likely be achieved at higher concentrations. Supporting this, recent studies have reported that rhein (1,8-dihydroxyanthraquinone-3-carboxylic acid) and DHDNA also exhibited bacteriostatic effects on *S. aureus* and *E. faecalis* at concentrations of 1×MIC and 2×MIC [8,35].

Some natural compounds exert their antimicrobial effect by permeabilizing membranes. Anthraquinone and its derivatives, being hydrophobic compounds, are traditionally believed to reduce the hydrophobic interactions between hydrocarbon chains in the phospholipid bilayer. This reduction weakens the cell membrane’s fluidity, increases its permeability, and ultimately disrupts the biological membrane’s structure [34]. We evaluated the increase in membrane permeability in strains exposed to increasing concentrations of HC (1×MIC, 2×MIC, 4×MIC). After 30 min of incubation, there was no significant increase in membrane permeability in the tested strains. However, after 6 h of incubation with 4×MIC, the membrane permeabilization rates reached 50%, 29%, and 59% for *E. faecalis*, *C. albicans*, and *S. epidermidis*, respectively. For *S. aureus*, the highest permeabilization rate of 79% was achieved after 24 h of incubation with 4×MIC. These results are in accordance with the permeabilization effects exerted by other anthraquinones, such as emodin, barbaloin, and 1,8-dihydroxy-anthraquinone (DAN), which interact with the cell envelope, increasing membrane permeability and causing leakage of intracellular contents, leading to cell death [44,45,46].

Bacterial biofilms are microbial communities that adhere to surfaces, are encapsulated in a self-produced extracellular matrix, and exhibit greater resistance to harsh environments, antimicrobial agents, and mechanical stresses compared to their planktonic forms [47,48]. Biofilm development occurs in four stages: initial adhesion, proliferation, maturation, and dispersion [49]. Initially, planktonic cells reversibly attach to a solid surface and begin to divide, accumulate, and form microcolonies [50]. With adequate nutrients, the biofilm enters the maturation stage, characterized by intercellular aggregation and the development of a complex three-dimensional structure [49]. For example, during the biofilm formation of *C. albicans* from 0 to 11 h, cells adhere to the surface of the carrier, begin to proliferate, and agglomerate. The cells then gather into groups and secrete an extracellular matrix, forming hyphae until reaching the mature biofilm stage [51]. Biofilm formation is considered one of the most important pathogenic factors of *C. albicans* and *S. aureus*, which is particularly concerning in the medical field due to numerous outbreaks of nosocomial infections [52,53]. Given the importance of biofilm structure, we tested the effect of HC on the forming biofilm and against a mature biofilm of the tested strains that were shown to produce biofilm. HC inhibited the formation of *S. aureus*, *E. faecalis*, and *C. albicans* in a non-dose-dependent manner. The reduction rate was comparable between MIC and 2×MIC for all strains, with a maximum reduction of 73% for *S. aureus*, 54% for *C. albicans*, and 34% for *E. faecalis*. 1×MIC was ineffective against mature biofilms of each strain; however, 2×MIC and 4×MIC led to a dose-dependent reduction, with a maximum reduction of 85% for *S. aureus*, 57% for *C. albicans*, and 65% for *E. faecalis*. These results agree with the known antibiofilm effects exerted by anthraquinones. For instance, alizarin (at 10 mg/L) decreased biofilm formation of *S. aureus* strains by ≥90%, while aloe-emodin at MIC (16 mg/L) affected biofilm formation but did not impact mature biofilms, which required a 2×MIC concentration (32 mg/L) [38,50]. Rhein also reduced mature biofilm biomass in a dose-dependent manner, achieving about 50% eradication at a dose of 50 µg/mL [35]. Additionally, alizarin and chrysazin, other natural anthraquinones, at 2 μg/mL dramatically reduced biofilm formation by *C. albicans* strains by ≥90%, while HC required higher concentrations (67 mg/L) to achieve lower inhibition rates [54]. Concerning *C. albicans*, the transition from yeast to hyphal phase is crucial for biofilm formation, with hyphal morphology being a key virulence factor [14]. Therefore, inhibiting both this transition and hyphal formation could effectively reduce biofilm-related infections caused by *C. albicans* [55]. In this study, the effect of HC on hyphae development after 6 and 24 h of incubation at concentrations of 0.5×MIC, 1×MIC, and 2×MIC was examined. Even at 0.5×MIC (8.3 mg/L), HC inhibited hyphae formation, which is a lower concentration compared to the effective doses of other anthraquinones like 2-alkyl anthraquinone (10 mg/L) and purpurin (20 mg/L). Therefore, at a concentration lower than 1×MIC, HC strongly decreased hyphal formation, demonstrating its potential anti-factors of virulence [13,37]. The hallachrome chemical structure and the structure of the other anthraquinones cited to compare the antibacterial effects are reported in Figure S2.

To fully understand the antimicrobial potential of Hallachrome, it will be essential to evaluate its activity against antibiotic-resistant clinical strains as well. Moreover, this study is pioneering in evaluating the antimicrobial activity of the only known anthraquinone of animal origin, which has a structure distinct from the more commonly studied anthraquinones.

## 4. Materials and Methods

### 4.1. H. parthenopeia Maintenance and Hallachrome Collection

The specimens of *H. parthenopeia* used in this experiment were collected from sandy-muddy infralittoral sea bottoms (3–10 m depth) off the coast of Marseille, France, and were acquired from a retailer, as in our previous studies [21,29,56]. After acclimatization, the healthy worms were individually housed in transparent polyethylene (PET) containers within a recirculating aquarium system, maintained according to the detailed procedures outlined by Ferri and colleagues [29]. To obtain the purple mucus, the worms were removed from the aquarium and placed in 20-liter high-density polyethylene (HDPE) flat tanks (60 × 40 cm) filled with approximately 7 cm of artificial seawater. They were then manually stimulated with a pin to induce the emission of defensive mucus. The freshly collected mucus was subsequently homogenized. After homogenization, Hallachrome (HC thereafter) was extracted and purified following the methods described by Simonini and colleagues [21], with some adaptations. The mucus was poured with dichloromethane (DCM) (2:1 ratio) in a separating funnel and left overnight. The DCM was then collected, replaced with a fresh dose, and left for an additional 10 h. Afterward, all the DCM was collected and evaporated, leaving a red row extract. The extract was then subjected to silica gel chromatography (0.06–0.20 mM, Carlo Erba reagents) using a 4:1 toluene-ethyl acetate elution mixture. On concentration, the red band gave purple crystals. High performance liquid chromatography analyses (Agilent 1200 series, Agilent Technologies, Santa Clara, USA) confirmed the purity of HC (procedures outlined in detail by Simonini and colleagues [21]. Aliquots of pure HC were then stored in a dark and dry place until use. The average amount of purple mucus emitted by each worm after mechanical stimulation was about 130 mL, corresponding to 2.3 mg of pure HC. The collection and purification of hallachrome are described in Figure S1.

### 4.2. Microbial Strains, Cultivation, and Chemicals Used in Testing

Given technical constraints, this first screening of hallachrome antimicrobial activity was performed using only classified microorganisms (ATCC-American Type Culture Collection), including *Enterococcus faecalis* ATCC 29212, *Staphylococcus aureus* ATCC 6538, *Staphylococcus epidermidis* ATCC 12228, *Pseudomonas aeruginosa* ATCC 9027, *Escherichia coli* ATCC 25922, and *Candida albicans* ATCC 10231. All strains were confirmed by matrix-assisted laser desorption ionization (MALDI) time-of-flight mass spectrometry (TOF/MS) and maintained in Tryptic Soy Broth (TSB, Oxoid S.p.A, Milan, Italy) containing 20% (*w*/*v*) glycerol at −80 °C until use.

The purified hallachrome was always dissolved in 100% dimethyl sulfoxide (DMSO) and diluted to reach 0.1% (vol/vol) DMSO in any experiment.

### 4.3. Antibacterial Susceptibility Testing and MIC Determination

The preliminary determination of HC activity against all tested bacteria was carried out using the agar disk diffusion assay, following the standard procedure of the Clinical and Laboratory Standards Institute (CLSI) [57]. Tryptic Soy Agar (TSA, Oxoid S.p.A, Milan, Italy) plates were seeded with 100 µL of 10^6^ CFU/mL cell suspensions, and then sterile disks of 6 mM in diameter, containing 10 µL of 2 mM HC (treatment), were placed on these plates. Higher concentration of HC resulted in its precipitation in aqueous medium (authors personal observations). After incubation at 37 °C or 30 °C (only for C. albicans) for 24 h, the antibacterial activity of the HC was assessed by measuring the diameters of the clear zone of inhibition around the disks. Only microorganisms for which the inhibition zone of the treatment was larger than those of the controls (1% DMSO) were considered susceptible to HC.

According to CLSI guidelines (2019) [57], the MIC values of HC were determined against all susceptible microorganisms using the broth microdilution method in 96-well microplates. In each well of a sterile 96-well microplate, 178 µL of TSB and 20 µL of bacterial suspensions were added, resulting in a final inoculum concentration of 10^6^ CFU/mL. Then, 2 µL of HC serial dilutions were added to obtain concentrations ranging from 2 to 0.015 mM. Negative control wells consisted of bacteria in TSB and bacteria in TSB added with 1% DMSO. Plates were mixed on a plate shaker at 300 rpm for 20 s and incubated at 37 °C or 30 °C for 24 h. The MIC was defined as the lowest concentration of HC that inhibited more than 80% of the growth of the tested microorganisms when the optical density (OD) was measured at 570 nm using a microtiter plate reader. Blank wells (TSB added with HC at different concentrations) were also included because HC alone absorbed light at a wavelength of 570 nm. All experiments were conducted in triplicate, and the results were expressed as the arithmetic mean of the three microtiter determinations.

### 4.4. Time-Kill Studies

In a 96-well sterile microplate, 190 µL of sterile nutrient broth and 10 µL of the susceptible strains, determined following the tests above, were placed in each well from a stock dilution to obtain a density of about 10^5^ CFU/mL. HC was added at different concentrations depending on the results obtained from the MIC determinations. For each strain, we tested at least the 2×MIC, 1×MIC, and 0.5×MIC. Negative control wells (microorganisms in TSB and microorganisms in TSB added with 1% DMSO) and blank wells (TSB added with HC at different concentrations) were set up. The microplate was incubated at 37 °C or 30 °C with an oscillating speed of 150 rpm, and the optical density (OD) was determined at 570 nm for bacteria and 540 nm for C. albicans at 1 h intervals for 24 h of exposure using an automatic microplate reader (Sunrise Tecan, Grödig, Austria). All experiments were conducted in triplicate, and the results were expressed as the arithmetic mean of the three determinations minus the average value of the respective blank for the HC treated.

### 4.5. Determination of Membrane Permeability Alteration via a Cristal Violet Assay

Alteration of membrane permeability was detected via a crystal violet assay [58]. Bacterial suspensions were grown in 2 mL of TSB at 37 °C or 30 °C for 24 h, then centrifuged at 4500× *g* for 5 min at 4 °C, washed twice, and resuspended in PBS (Phosphate Buffer Saline, pH 7.4). HC was added at 0.5×MIC, 1×MIC, 2×MIC, and 4×MIC concentrations for each susceptible strain. The suspensions were incubated at 37 °C or 30 °C for 30 min, 6 h, or 24 h. Negative controls (microorganisms in TSB and microorganisms in TSB added with 1% DMSO) and blanks (TSB added with HC at different concentrations) were set up. The cells were centrifuged at 9300× *g* for 5 min, resuspended in PBS containing 5 µg/mL of crystal violet, and incubated for 10 min at 37 °C. After incubation, the suspensions were centrifuged at 13,400× *g* for 15 min, and their optical density (OD) was measured at 590 nm using a microplate reader (Sunrise Tecan, Grödig, Austria). All experiments were conducted in triplicate, and results were expressed as the arithmetic mean of the three determinations minus the average value of the respective blank for the HC-treated samples. The percentage of crystal violet absorbed was calculated using the following formula: (OD value of sample)/(OD value of 5 µg/mL crystal violet solution) × 100.

### 4.6. Determination of HC Activity on Biofilm

The capability of all tested strains to form biofilm was studied using a modified 96-well microtiter plates method [59]. Only for the strains that demonstrated particularly strong biofilm-forming abilities were the effects of HC on the mature and the formation biofilm evaluated. For each microorganism, an overnight culture (18–24 h) was diluted in fresh sterile TSB to a final concentration of 10^6^ CFU/mL, and 200 µL was dispensed into each well of a 96-well plate and incubated for 24 h at 37 °C or 30 °C. To evaluate the activity against mature biofilm, the medium was gently aspirated to remove planktonic, and the wells were washed three times with sterile phosphate-buffered saline (PBS, pH 7.2). HC was added at 1×MIC, 2×MIC, and 4×MIC concentrations. The plates were then incubated for 24 h at 37 °C or 30 °C. For the determination of the effectiveness on biofilm formation, after an incubation time of 6 h at 37 °C or 30 °C, HC was added at 1×MIC, 2×MIC, and 4×MIC concentrations. Subsequently, the plates were incubated for another 24 h at 37 °C or 30 °C. Microorganisms in TSB and microorganisms in TSB added with 1% DMSO were set up as negative controls. Following the additional incubation, biofilm biomass was quantified using the crystal violet staining method described by Stepanovic and colleagues [60]. After incubation, the plates were washed three times with sterile PBS (pH 7.2) to remove planktonic bacteria and fixed with 150 µL of methanol for 15 min. The plates were then emptied, air-dried, and stained with 150 µL of crystal violet (2% Hucker crystal violet) for 5 min at room temperature. After staining, the wells were washed three times with milli-Q water (mqH20), and the dye bound to the cells was dissolved in 33% glacial acetic acid (Sigma-Aldrich, Saint Louis, MO, USA). Results were expressed as optical density (OD) values at 595 nm using a microplate reader (Sunrise Tecan, Grödig, Austria). The arithmetic mean of the three determinations was calculated, and the standard deviation was reported as error bars.

### 4.7. Determination of HC Activity on Biofilm: Microscopic Study

The effectiveness of HC treatments on the mature and on the formation biofilm was also evaluated with a morphological study using a light microscope. Both the mature and the formation biofilm were tested as described above. Briefly, an overnight culture (18–24 h) of each biofilm-producing strain was diluted in fresh sterile TSB to a final concentration of 10^6^ CFU/mL, and 1 mL was dispensed into each well of a 12-well polystyrene plate to have a larger optical field to observe under a microscope. After 24 h, for the mature biofilm, the medium was gently aspirated to remove planktonic bacteria, and the wells were washed three times with sterile PBS. Then, HC was added at the MIC concentration, and the microtiter plate was incubated for 24 h at 37 °C or 30 °C. While, for the determination of the effectiveness on biofilm formation, after an incubation time of 6 h at 37 °C or 30 °C, HC was added at MIC, 2×MIC, and 4×MIC concentrations. Subsequently, the plates were incubated for another 24 h at 37 °C or 30 °C. Microorganisms in TSB and microorganisms in TSB added with 1% DMSO were set up as negative controls. Following the additional incubation, both the HC-treated samples and the negative controls were washed three times with sterile PBS and fixed with 150 µL of methanol for 5 min. The supernatant was then removed, and 150 µL of crystal violet (CV) solution at 0.1% was added to each well. After incubation at room temperature for 30 min, the excess CV was removed by washing three times with sterile PBS. The plates were then observed under a light microscope, the Nikon Eclipse TS 100 (Nikon Instruments Inc., Melville, NY, USA). Samples were photographed with a DS-2Mv Nikon digital camera (Nikon Inc., Melville, NY, USA).

### 4.8. Determination of HC Activity on Mature Biofilm by Fluorescence Microscopy Assay

The effect of HC on mature biofilm formation was evaluated in a 6-well microtiter plate, as described above. Briefly, an overnight culture (18–24 h) of the best biofilm-producing tested strains was diluted in fresh sterile TSB to a final concentration of 10^6^ CFU/mL, and 2 mL was dispensed into each well of a 6-well polystyrene plate. After incubation for 24 h at 37 °C or 30 °C, each well was washed twice with PBS, and fresh TSB with HC at MIC was added. Microorganisms in TSB and microorganisms in TSB added with 1% DMSO were set up as negative controls. Subsequently, an additional incubation at 37 °C or 30 °C for 24 h, wells were washed twice with PBS to remove all planktonic forms. Biofilm was fixed for 30 min with PBS-buffered 4% paraformaldehyde, then the samples were washed twice with PBS, treated with Prolong Gold antifade (PLGAR) (Thermo Fisher Scientific, Walthan, MA, USA), and stained by the “live/dead cells stain kit” (Thermo Fisher Scientific, Waltham, MA, USA), according to manufacturer instructions. The method is based on the use of propidium iodide (PI) as a marker of dead cells and 5(6)-carboxyfluorescein diacetate (cFDA) to detect alive cells. After incubation in the dark at room temperature for 30 min, biofilm was visualized by epifluorescence microscopy on a Nikon Eclipse 90i imaging system equipped with Normaski DIC optics (Nikon Instruments Inc., Melville, NY, USA). Samples were photographed with a DS-2Mv Nikon digital camera.

### 4.9. C. albicans Hyphae Formation

Hyphal growth assay of *C. albicans* was performed in Yeast Peptone Dextrose Broth (YPD, Oxoid S.p.A, Milan, Italy) supplemented with 10% fetal bovine serum (FBS). To assess the effect of HC in liquid medium, a mixture of 990 μL *C. albicans* suspensions (10^6^ CFU/mL) and 10 μL of 25×MIC, 50×MIC, 100×MIC and 200×MIC hallachrome concentrations was added into each well of a sterile 12-well microplate and aerobically incubated at 30 °C for 6 and 24 h. Inhibition of the yeast-to-hyphae transition was evaluated under a light microscope, the Nikon Eclipse 90i imaging system equipped with Nomarski DIC optics (Nikon Instruments, Melville, NY, USA). The DS-2Mv Nikon digital camera was employed to obtain images [61].

### 4.10. Statistical Analysis

Each experiment was replicated three times. Statistical significance was determined using *t*-tests and ANOVA, followed by Tukey’s post hoc test, performed with the statistical software PAST4.0. Differences were considered significant at a *p*-value ≤ 0.05. The kinetic curves of the raw data for the various conditions were compared to the untreated control for the area under the curve (AUC) using GraphPad Prism 10 software. Growth kinetics were also analyzed by fitting the data to a sigmoid model $\left(y=L/(1+e^{\left(-k(x-x0)\right)})+b\right)$ to determine the time at which 50% of maximal growth occurred under each experimental condition.

## 5. Conclusions

This study marks the first antimicrobial activity tests conducted on Hallachrome, the only anthraquinone derived from an animal source, thereby introducing a novel area of research in marine-derived bioactive compounds. The Hallachrome exhibits notable antimicrobial properties, particularly against Gram-positive bacteria and the human fungal pathogen *Candida albicans*. Its ability to permeabilize cell membranes and inhibit hyphal formation at concentrations lower than the MIC, coupled with its effectiveness in preventing both the formation and maturation of biofilms, highlights its therapeutic potential. These findings align with the known effects of other anthraquinones, underscoring the promise of marine organisms as valuable sources for new natural-derived antimicrobial compounds. This underexplored anthraquinone deserves further research, first to evaluate Hallachrome’s efficacy against clinically isolated bacteria. Such studies could reveal whether Hallachrome is more or less effective against these clinically relevant strains, ultimately guiding its potential development as a therapeutic agent. This study paved the way for the development of conjugated forms with other compounds to enhance its antimicrobial efficacy and delivery mechanisms, thereby significantly improving its practical application.

## Figures and Tables

**Table 1:** Minimum inhibitory concentration of HC active on tested strains (*C. albicans* ATCC 10231, *E. faecalis* ATCC 29212, *S. aureus* ATCC 6538, *S. epidermidis* ATCC 12228, *P. aeruginosa* ATCC 9027, and *E. coli* ATCC 25922), and their inhibition rate.

Bacterial Strain	MIC (mM)	Inhibition Rate
*Candida albicans* ATCC 10231	0.06	97%
*Enterococcus faecalis* ATCC 29212	0.25	88%
*Staphylococcus aureus* ATCC 6538	0.25	92%
*Staphylococcus epidermidis* ATCC 12228	0.125	98%
*Pseudomonas aeruginosa* ATCC9027	>2	0%
*Escherichia coli* ATCC25922	>2	0%

## Data Availability

The data are contained within the article and/or are available from the corresponding author upon reasonable request.

## Supplementary Materials

The following supporting information can be downloaded at: https://www.mdpi.com/article/10.3390/md22090380/s1, Figure S1: representation of hallachrome collection and purification protocol; Figure S2: Chemical structure of Hallachrome and the other anthraquinones cited in the text.

## References

[1] Salam M.A., Al-Amin M.Y., Salam M.T., Pawar J.S., Akhter N., Rabaan A.A., Alqumber M.A.A. (2023). Antimicrobial Resistance: A Growing Serious Threat for Global Public Health. Healthcare.

[2] WHO World Antimicrobial Awareness Week-WAAW from 18 to 24 November 2020. https://www.who.int/campaigns/world-antimicrobial-awareness-week/2020.

[3] Spellberg B., Powers J.H., Brass E.P., Miller L.G., Edwards J. (2004). Trends in Antimicrobial Drug Development: Implications for the Future. Clin. Infect. Dis..

[4] López Y., Cepas V., Soto S.M., Rampelotto P., Trincone A. (2018). The Marine Ecosystem as a Source of Antibiotics. Grand Challenges in Marine Biotechnology.

[5] Chinemerem Nwobodo D., Ugwu M.C., Oliseloke Anie C., Al-Ouqaili M.T., Chinedu Ikem J., Victor Chigozie U., Saki M. (2022). Antibiotic Resistance: The Challenges and Some Emerging Strategies for Tackling a Global Menace. J. Clin. Lab. Anal..

[6] Vivas R., Barbosa A.A.T., Dolabela S.S., Jain S. (2019). Multidrug-Resistant Bacteria and Alternative Methods to Control Them: An Overview. Microb. Drug Resist..

[7] Iseppi R., Mariani M., Condò C., Sabia C., Messi P. (2021). Essential Oils: A Natural Weapon against Antibiotic-Resistant Bacteria Responsible for Nosocomial Infections. Antibiotics.

[8] Amorim J., Vásquez V., Cabrera A., Martínez M., Carpio J. (2024). In Silico and In Vitro Identification of 1,8-Dihydroxy-4,5-dinitroanthraquinone as a New Antibacterial Agent against *Staphylococcus aureus* and *Enterococcus faecalis*. Molecules.

[9] Sardi J.C., Scorzoni L., Bernardi T., Fusco-Almeida A.M., Mendes Giannini M.J. (2013). Candida Species: Current Epidemiology, Pathogenicity, Biofilm Formation, Natural Antifungal Products and New Therapeutic Options. J. Med. Microbiol..

[10] Zida A., Bamba S., Yacouba A., Ouedraogo-Traore R., Guiguemdé R.T. (2017). Anti-*Candida albicans* Natural Products, Sources of New Antifungal Drugs: A Review. J. Mycol. Med..

[11] Ma W., Liu C., Li J., Hao M., Ji Y., Zeng X. (2020). The Effects of Aloe Emodin-Mediated Antimicrobial Photodynamic Therapy on Drug-Sensitive and Resistant *Candida albicans*. Photochem. Photobiol. Sci..

[12] Mayer F.L., Wilson D., Hube B. (2013). *Candida albicans* Pathogenicity Mechanisms. Virulence.

[13] Song Y., Wang Z., Long Y., Mao Y., Jiang F., Lu Y. (2022). 2-Alkyl-anthraquinones Inhibit *Candida albicans* Biofilm via Inhibiting the Formation of Matrix and Hyphae. Res. Microbiol..

[14] Shin D.S., Eom Y.B. (2019). Zerumbone Inhibits *Candida albicans* Biofilm Formation and Hyphal Growth. Can. J. Microbiol..

[15] Newman D.J., Cragg G.M. (2007). Natural Products as Sources of New Drugs Over the Last 25 Years. J. Nat. Prod..

[16] Hughes C.C., Fenical W. (2010). Antibacterials from the Sea. Chemistry.

[17] Thawabteh A.M., Swaileh Z., Ammar M., Jaghama W., Yousef M., Karaman R., Bufo S.A., Scrano L. (2023). Antifungal and Antibacterial Activities of Isolated Marine Compounds. Toxins.

[18] Struck T., Paul C., Hill N., Hartmann S., Hosel C., Kube M., Lieb B., Meyer A., Tiedmannm R., Purschke G. (2011). Phylogenomic Analyses Unravel Annelid Evolution. Nature.

[19] Rodrigo A.P., Costa P.M. (2019). The Hidden Biotechnological Potential of Marine Invertebrates: The Polychaeta Case Study. Environ. Res..

[20] Coutinho M.C.L., Teixeira V.L., Santos C.S.G. (2018). A Review of “Polychaeta” Chemicals and Their Possible Ecological Role. J. Chem. Ecol..

[21] Simonini R., Iori D., Forti L., Righi S., Prevedelli D. (2019). Ecotoxicity of Hallachrome, an Unusual 1-2 Anthraquinone Excreted by the Infaunal Polychaete *Halla parthenopeia*: Evidence for a Chemical Defence?. Invertebr. Surviv. J..

[22] Macedo M.W.F.S., Cunha N.B., Carneiro J.A., Costa R.A., Alencar S.A., Cardoso M.H., Franco O.L., Dias S.C. (2021). Marine Organisms as a Rich Source of Biologically Active Peptides. Front. Mar. Sci..

[23] Cuvillier-Hot V., Boidin-Wichlacz C., Tasiemski A. (2014). Polychaetes as Annelid Models to Study Ecoimmunology of Marine Organisms. J. Mar. Sci. Technol..

[24] Rajanbabu V., Chen J.Y., Wu J.L. (2015). Antimicrobial Peptides from Marine Organisms. Springer Handbook of Marine Biotechnology.

[25] Chain B.M., Anderson R.S. (1983). Antibacterial of the Coelomic Fluid from the Polychaeta, *Glycera dibranchiata*. Partial Purification and Biochemical Characterization of the Active Factor. Biol. Bull..

[26] Higa T., Scheuer P.J. (1974). Thelepin, a New Metabolite from the Marine Annelid *Thelepus setosus*. J. Am. Chem. Soc..

[27] Stabili L., Licciano M., Giangrande A., Gerardi C., De Pascali S.A., Fanizzi F.P. (2019). First Insight on the Mucus of the Annelid *Myxicola infundibulum* (Polychaeta, Sabellidae) as a Potential Prospect for Drug Discovery. Mar. Drugs.

[28] Prota G., D’Agostino M., Misuraca G. (1972). The Structure of Hallachrome: 7-Hydroxy-8-methoxy-6-methyl-1, 2-Anthraquinone. J. Chem. Soc. Perkin Trans..

[29] Ferri A., Righi S., Prevedelli D., Simonini R. (2024). Optimal Growth and Feeding Behaviour of the Valuable Bait *Halla parthenopeia* (Polychaeta: Oenonidae) in Small-Scale Aquaculture. Aquac. Res..

[30] Ferri A., Costa P.M., Simonini R. (2024). Unveiling the Microanatomy of Secretory Organs in *Halla parthenopeia* (Oenonidae): Implications for the Feeding Strategies and Defence Mechanisms of a Carnivorous Burrowing Polychaete. J. Anat..

[31] Diaz-Munoz G., Miranda I.L., Sartori S.K., De Rezende D.C., Diaz M.A. (2018). Anthraquinones: An Overview. Stud. Nat. Prod. Chem..

[32] Malmir M., Serrano R., Silva O., Antonio M.V. (2017). Anthraquinones as Potential Antimicrobial Agents—A Review. Antimicrobial research: Novel bioknowledge and educational programs.

[33] Friedman M., Xu A., Lee R., Nguyen D.N., Phan T.A., Hamada S.M., Panchel R., Tam C.C., Kim J.H., Cheng L.W. (2020). The Inhibitory Activity of Anthraquinones against Pathogenic Protozoa, Bacteria, and Fungi and the Relationship to Structure. Molecules.

[34] Qun T., Zhou T., Hao J., Wang C., Zhang K., Xu J., Zhou W. (2023). Antibacterial Activities of Anthraquinones: Structure–Activity Relationships and Action Mechanisms. RSC Med. Chem..

[35] Dell’Annunziata F., Folliero V., Palma F., Crudele V., Finamore E., Sanna G., Manzin A., De Filippis A., Galdiero M., Franci G. (2022). Anthraquinone Rhein Exhibits Antibacterial Activity against *Staphylococcus aureus*. Appl. Sci..

[36] Farooq U., Khan S., Naz S., Khan A., Khan A., Ahmed A., Khan A.R. (2017). Three New Anthraquinone Derivatives Isolated from *Symplocos racemosa* and Their Antibiofilm Activity. Chin. J. Nat. Med..

[37] Duraipandiyan V., AL-Dhabi N.A., Balachandran C., Raj M.K., Arasu M.V., Ignacimuthu S. (2014). Novel 1,5,7-Trihydroxy-3-Hydroxy Methyl Anthraquinone Isolated from Terrestrial Streptomyces sp. (eri-26) with Antimicrobial and Molecular Docking Studies. Appl. Biochem. Biotechnol..

[38] Lee J.H., Kim Y.G., Ryu Y., Lee J. (2016). Calcium-Chelating Alizarin and Other Anthraquinones Inhibit Biofilm Formation and the Hemolytic Activity of *Staphylococcus aureus*. Sci. Rep..

[39] Chen H., Du K., Sun Y.J., Hao Z.Y., Zhang Y.L., Bai J., Wang Q.H., Hu H.Y., Feng W.S. (2020). Solanrubiellin A, a Hydroanthraquinone Dimer with Antibacterial and Cytotoxic Activity from *Solanum lyratum*. Nat. Prod. Res..

[40] Zhang C., Ondeyka J.G., Zink D.L., Basilio A., Vicente F., Collado J., Singh S.B. (2009). Isolation, Structure and Antibacterial Activity of Pleosporone from a Pleosporalean Ascomycete Discovered by Using Antisense Strategy. Bioorg. Med. Chem..

[41] Janeczko M., Masłyk M., Kubiński K., Golczyk H. (2017). Emodin, a Natural Inhibitor of Protein Kinase CK2, Suppresses Growth, Hyphal Development, and Biofilm Formation of *Candida albicans*. Yeast.

[42] Kang K., Fong W.P., Tsang P.W. (2010). Novel Antifungal Activity of Purpurin against Candida Species in vitro. Med. Mycol..

[43] Chukwujekwu J.C., Coombes P.H., Mulholland D.A., Van Staden J. (2006). Emodin, an Antibacterial Anthraquinone from the Roots of *Cassia occidentalis*. S. Afr. J. Bot..

[44] Alves D.S., Pérez-Fons L., Estepa A., Micol V. (2004). Membrane-Related Effects Underlying the Biological Activity of the Anthraquinones Emodin and Barbaloin. Biochem. Pharmacol..

[45] Raghuveer D., Pai V.V., Murali T.S., Nayak R. (2023). Exploring Anthraquinones as Antibacterial and Antifungal Agents. ChemistrySelect.

[46] Wei Y., Liu Q., Yu J., Feng Q., Zhao L., Song H., Wang W. (2014). Antibacterial Mode of Action of 1,8-Dihydroxy-Anthraquinone from *Porphyra haitanensis* against *Staphylococcus aureus*. Nat. Prod. Res..

[47] Flemming H.C., Wingender J. (2010). The Biofilm Matrix. Nat. Rev. Microbiol..

[48] Hoiby N., Bjarnsholt T., Givskov M., Molin S., Ciofu O. (2010). Antibiotic Resistance of Bacterial Biofilms. Int. J. Antimicrob. Agents.

[49] Otto M. (2008). Staphylococcal Biofilms. Curr. Top. Microbiol. Immunol..

[50] Xiang H., Cao F., Ming D., Zheng Y., Dong X., Zhong X., Mu D., Li B., Zhong L., Cao J. (2017). Aloe-Emodin Inhibits *Staphylococcus aureus* Biofilms and Extracellular Protein Production at the Initial Adhesion Stage of Biofilm Development. Appl. Microbiol. Biotechnol..

[51] Chandra J., Kuhn D.M., Mukherjee P.K., Hoyer L.L., McCormick T., Ghannoum M.A. (2001). Biofilm Formation by the Fungal Pathogen *Candida albicans*: Development, Architecture, and Drug Resistance. J. Bacteriol..

[52] Park S.J., Han K.H., Park J.Y., Choi S.J., Lee K.H. (2014). Influence of Bacterial Presence on Biofilm Formation of *Candida albicans*. Yonsei Med. J..

[53] Taylor T.A., Unakal C.G. (2023). Staphylococcus Aureus Infection.

[54] Manoharan R.K., Lee J.H., Kim Y.G., Lee J. (2017). Alizarin and Chrysazin Inhibit Biofilm and Hyphal Formation by *Candida albicans*. Front. Cell. Infect. Microbiol..

[55] Gauwerky K., Borelli C., Korting H.C. (2009). Targeting Virulence: A New Paradigm for Antifungals. Drug Discov. Today.

[56] Iori D., Forti L., Massamba-N’Siala G., Prevedelli D., Simonini R. (2014). Toxicity of the Purple Mucus of the Polychaete *Halla parthenopeia* (Oenonidae) Revealed by a Battery of Ecotoxicological Bioassays. Sci. Mar..

[57] (2019). CLSI Performance Standards for Antimicrobial Susceptibility Testing.

[58] Devi K.P., Nisha S.A., Sakthivel R., Pandian S.K. (2010). Eugenol (An Essential Oil of Clove) Acts as an Antibacterial Agent against *Salmonella typhi* by Disrupting the Cellular Membrane. J. Ethnopharmacol..

[59] Hemaiswarya S., Kruthiventi A.K., Doble M. (2008). Synergism between Natural Products and Antibiotics against Infectious Diseases. Phytomedicine.

[60] Stepanovic S., Vukovic D., Dakic I., Savic B., Svabic-Vlahovic M. (2000). A Modified Microtiter-Plate Test for Quantification of Staphylococcal Biofilm Formation. J. Microbiol. Methods.

[61] Wang S., Wang Q., Yang E., Yan L., Li T., Zhuang H. (2017). Antimicrobial Compounds Produced by Vaginal *Lactobacillus crispatus* Are Able to Strongly Inhibit *Candida albicans* Growth, Hyphal Formation and Regulate Virulence-Related Gene Expressions. Front. Microbiol..

