# Supplementary material for: Exploring the Antimicrobial Potential of Hallachrome, a Defensive Anthraquinone from the Marine Worm Halla parthenopeia (Polychaeta)
Source: Mar Drugs. 2024 Aug 24;22(9):380. doi: 10.3390/md22090380 (PMC11433307; doi:10.3390/md22090380)
Supplement: Supplementary file 1 [file marinedrugs-22-00380-s001.zip › marinedrugs-3105131-supplementary.pdf]

FIGURE S1: representation of hallachrome collection and purification protocol.

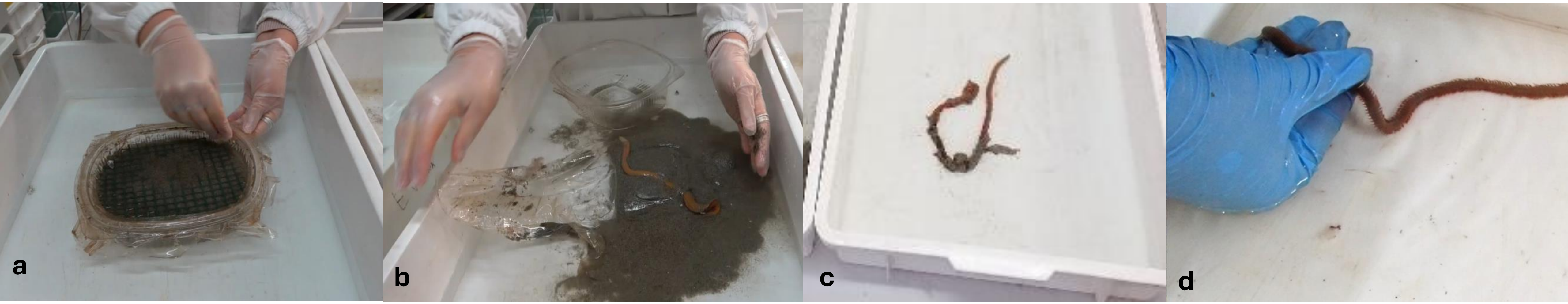

The containers in which the worms are kept (a) are disassembled, and the worms are gently removed from the sand (b). They are then transferred to a tank with clean water (c) and manually annoyed (d) until they secrete substantial amounts of purple mucus (e). The fresh mucus is collected with a syringe and mechanically homogenized in a beaker (f).

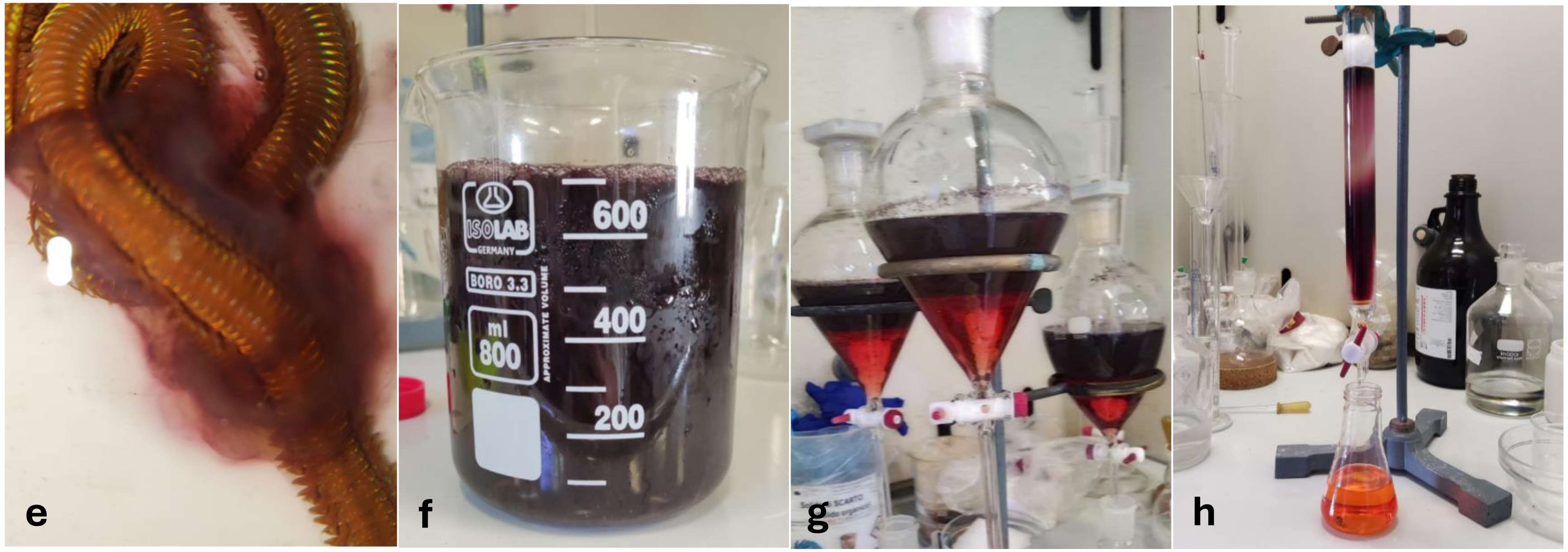

Next, the hallachrome is extracted through partitioning with dichloromethane in a separatory funnel (g). The crude hallachrome is then evaporated and redissolved in a small amount of dichloromethane. This solution is purified using a silica gel column with a 4:1 toluene-ethyl acetate eluent phase (h). The pure hallachrome is evaporated with a rotary evaporator.

FIGURE S2: Chemical structure of Hallachrome and the other anthraquinones cited in the text

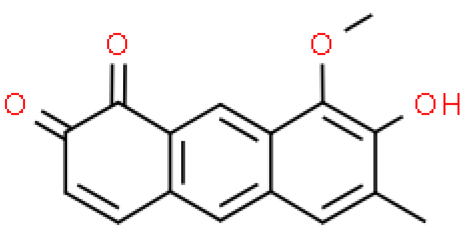

HALLACHROME

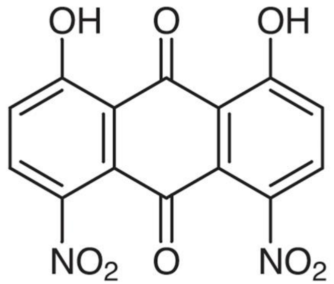

1,8-dihydroxy-4,5-dinitroanthraquinone (DHDNA)

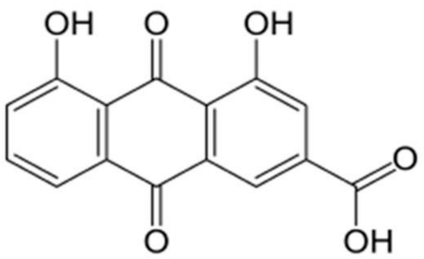

Rhein

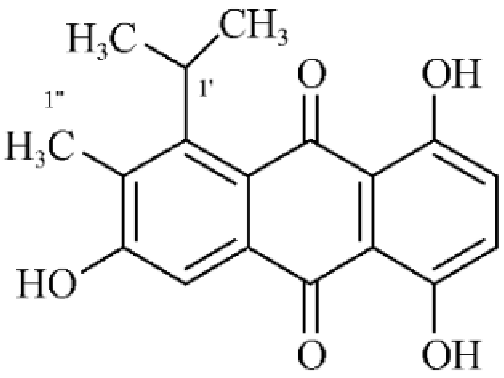

Symploquinone C

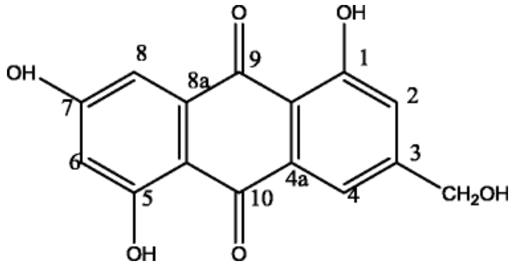

1,5,7-Trihydroxy-3-Hydroxy Methyl Anthraquinone

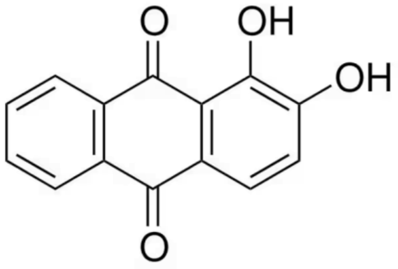

Alizarin

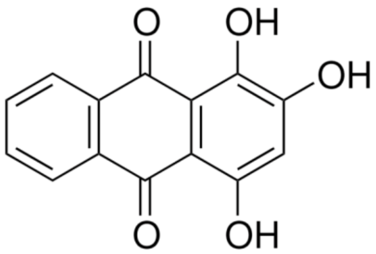

purpurin

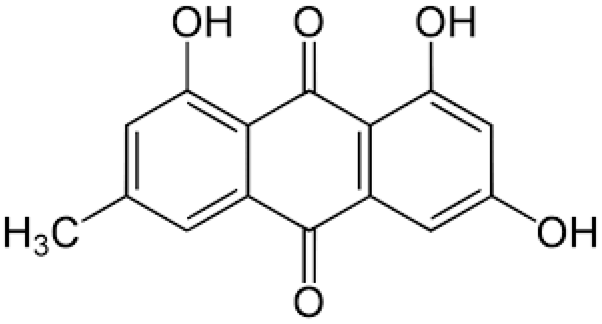

Emodin

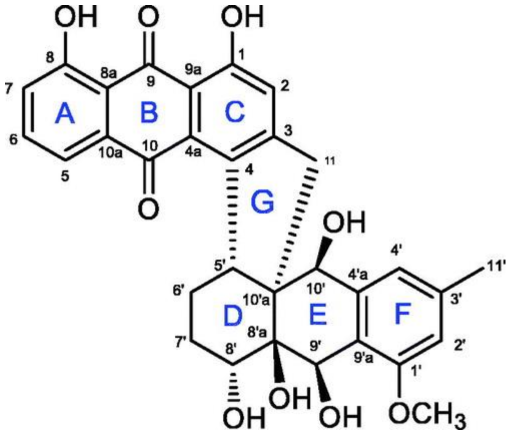

Solanrubiellin A

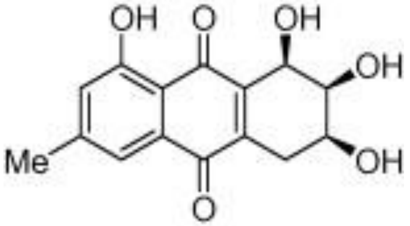

Pleosporone

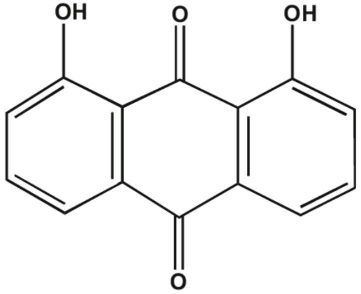

Chrysazin

Chemical structure of Hallachrome and the other anthraquinones cited in the text to compare the antibacterial effect.
